# Exergame and Balance Training Modulate Prefrontal Brain Activity during Walking and Enhance Executive Function in Older Adults

**DOI:** 10.3389/fnagi.2016.00066

**Published:** 2016-04-12

**Authors:** Patrick Eggenberger, Martin Wolf, Martina Schumann, Eling D. de Bruin

**Affiliations:** ^1^Institute of Human Movement Sciences and Sport, Department of Health Sciences and Technology, ETH ZurichZurich, Switzerland; ^2^Biomedical Optics Research Laboratory, Department of Neonatology, University Hospital ZurichZurich, Switzerland; ^3^Department of Epidemiology, CAPHRI School for Public Health and Primary Care, Maastricht UniversityMaastricht, Netherlands; ^4^Centre for Evidence Based Physiotherapy, Maastricht UniversityMaastricht, Netherlands

**Keywords:** prefrontal cortex, hemispheric asymmetry, functional near-infrared spectroscopy, cognition, gait, interactive cognitive-motor video game dancing, simultaneous cognitive-physical training, elderly

## Abstract

Different types of exercise training have the potential to induce structural and functional brain plasticity in the elderly. Thereby, functional brain adaptations were observed during cognitive tasks in functional magnetic resonance imaging studies that correlated with improved cognitive performance. This study aimed to investigate if exercise training induces functional brain plasticity during challenging treadmill walking and elicits associated changes in cognitive executive functions. Forty-two elderly participants were recruited and randomly assigned to either interactive cognitive-motor video game dancing (DANCE) or balance and stretching training (BALANCE). The 8-week intervention included three sessions of 30 min per week and was completed by 33 participants (mean age 74.9 ± 6.9 years). Prefrontal cortex (PFC) activity during preferred and fast walking speed on a treadmill was assessed applying functional near infrared spectroscopy pre- and post-intervention. Additionally, executive functions comprising shifting, inhibition, and working memory were assessed. The results showed that both interventions significantly reduced left and right hemispheric PFC oxygenation during the acceleration of walking (*p* < 0.05 or trend, *r* = 0.25–0.36), while DANCE showed a larger reduction at the end of the 30-s walking task compared to BALANCE in the left PFC [*F*_(1, 31)_ = 3.54, *p* = 0.035, *r* = 0.32]. These exercise training induced modulations in PFC oxygenation correlated with improved executive functions (*p* < 0.05 or trend, *r* = 0.31–0.50). The observed reductions in PFC activity may release cognitive resources to focus attention on other processes while walking, which could be relevant to improve mobility and falls prevention in the elderly. This study provides a deeper understanding of the associations between exercise training, brain function during walking, and cognition in older adults.

## Introduction

The human brain experiences a variety of prominent structural changes during the course of aging. The most apparent change includes a progressive brain atrophy, which is compensated for by increased ventricular spaces and cerebrospinal fluid. Shrinkage of the brain's size accelerates in old age and is gradually manifested from anterior to posterior regions. Thereby, the volume of prefrontal regions is affected in the most pronounced manner (Deary et al., 2009; Lockhart and DeCarli, 2014). Advancing age is also associated with a reduction of cognitive functioning, which is prevalent in almost every second elderly person (Scafato et al., 2010). However, the evidence appears not to be sufficient to conclude that altered brain structures reflect the neuroanatomical substrates for the age-related decline of cognitive performance (Fjell and Walhovd, 2010; Salthouse, 2011; Bennett and Madden, 2014). An important reason for this seemingly discrepancy is that the aging brain is able to counterbalance structural attenuations by altering the functional recruiting patterns and, thereby, maintaining cognitive functions (Deary et al., 2009; Grady, 2012). Such processes reflect functional brain and cognitive plasticity in the aging human brain.

Intriguingly, functional and structural brain plasticity has been observed in older adults after various types of physical exercise training, including cardiovascular, strength, coordination, and balance training, and was consistently correlated with improved cognitive performance (Voss et al., 2010, 2013; Erickson et al., 2011; Voelcker-Rehage et al., 2011; Liu-Ambrose et al., 2012; ten Brinke et al., 2015). Nonetheless, the functional brain adaptations were evident in simple cognitive tasks that can be performed during functional magnetic resonance imaging (fMRI) administration. This leaves the important question unanswered whether functional brain adaptations after exercise training could also be observed in real-life physical functioning of older adults, such as during walking? It was demonstrated with functional near-infrared spectroscopy (fNIRS) that brain activity is elevated during walking particularly in the prefrontal cortex (PFC), the premotor cortex, and in the supplementary motor area (Harada et al., 2009; Holtzer et al., 2014; Hamacher et al., 2015). Moreover, brain activity was further increased to maintain gait in circumstances with interference from additional secondary tasks (Holtzer et al., 2011; Lu et al., 2015) or under challenging walking situations (Suzuki et al., 2004; Koenraadt et al., 2014). Although, none of these studies assessed cognitive functioning, their findings of frontal and prefrontal brain areas being involved in walking tasks supports the theory that attention and executive functions, which are associated with these brain areas, play an important role in locomotion in older adults (Amboni et al., 2013). Emerging evidence indicates that aging-related decline in higher order cognitive processing, e.g., in attention and executive functioning, are associated with impaired gait in older adults (Yogev-Seligmann et al., 2008; de Bruin and Schmidt, 2010). It would, therefore, be of particular interest to understand brain-mediated effects of exercise training on improved mobility and cognition in order to define effective exercise programs for the elderly.

Exercise programs comprising simultaneous cognitive and motor training, such as video game dancing, complemented with conventional strength and balance training were shown to improve both, single- and dual-task gait parameters (Pichierri et al., 2012; Eggenberger et al., 2015b) as well as higher cognitive processing as measured by standard neuropsychological tests (Fraser et al., 2014; Eggenberger et al., 2015a). Also other interactive cognitive-motor step training games led to improvements in cognitive functions in older adults (Schoene et al., 2015). These findings support the results of a systematic review investigating the positive effects video games may have on cognition and brain structure (Shams et al., 2015). However, the assumption that exercise training induced alterations in frontal and prefrontal brain functioning could play a mediating role for gait and cognitive improvements warrants further investigation to be verified. First, since the literature on walking-related brain activity is sparse and mostly included small samples of young adults. Moreover, longitudinal interventions assessing exercise training induced changes in functional brain activity during walking and concomitant changes in cognitive performance are, to the best of our knowledge, lacking.

To date, it has not been shown that a cognitive-motor training based on a video game approach can improve brain functioning in frontal and prefrontal brain areas during walking. Therefore, the aim of this study was to compare the effects of cognitive-motor video game dancing against conventional balance training on PFC activity during walking and on executive functions. We hypothesized, (1) that cognitive-motor video game dancing would elicit larger training-induced reductions of PFC oxygenation during walking than conventional balance training and (2) that training-induced changes in PFC oxygenation would correlate with changes in executive functions.

## Materials and methods

### Study design and participants

This study was a randomized, controlled trial, including a two groups parallel 8-week training intervention. Assessments were performed pre- and post-training. Data collection and training were performed at Geriatrische Klinik St.Gallen, Switzerland. The study protocol was approved by the local ethics committee of the canton St.Gallen, Switzerland (study-number: EKSG 13/089) and registered at Current Controlled Trials under ISRCTN82949128. No changes were made to the planned methods after trial commencement. Our reporting adheres to the CONSORT 2010 guidelines (Moher et al., 2010).

Participants' recruitment lasted from September until November 2013. The first 8-week training block started in mid-October, and the second 8-week training block, with participants that were recruited later, started at the beginning of December 2013. Participants were recruited through a newspaper article, a local senior organization (Pro Senectute St.Gallen), senior residence facilities, and senior sports clubs. Interested persons were invited to an information event. We included male and female participants because both genders are similarly affected by age-related cognitive decline (Scafato et al., 2010). For eligibility, participants had to be older than 65 years of age, live independently or at senior residence facilities, and sign informed consent in accordance with the Declaration of Helsinki. Participants had to be able to walk for about 10 min on a treadmill. Residents of retirement homes classified 0, 1, or 2 within the Swiss classification system for health-care requirements (BESA-levels, German abbreviation for: Bewohner-Einstufungs- und Abrechnungs-System) could enroll in the study. Level 0 meaning the person does not need care or treatment; Level 1 to 2 meaning, the person only needs little care or treatment. Seniors with diagnosed Alzheimer's disease, dementia, or recent head injury were excluded. Judgment by their primary care physician was required in the case of acute or instable chronic diseases (e.g., stroke, diabetes) and rapidly progressing or terminal illnesses before accepting a person for participation.

A priori power analysis was performed with the G^*^Power 3.1.3 Software (Faul et al., 2007) and revealed a sample size of 34 participants in order to achieve 80% power for a two-groups pre- and post-test design (17 participants per group). The α-level was set at 0.05 and the effect size *f* at 0.25. For the two-groups randomization scheme, a random number between zero and 100 was generated for each participant with Microsoft Excel software. Thereafter, the lower half of random numbers/participants was assigned to the video game dance group and the upper half to the balance and stretching group. Participants were blinded to the expected study outcome, while blinding of the investigators was not possible since they supervised and conducted training and testing sessions.

### Training interventions

Three 30-min training sessions per week were performed on separate days (Monday, Wednesday, Friday) for 8 weeks. Groups of four participants were instructed by two trained postgraduate students. Training programs adhered to the current recommendations for physical fitness and fall prevention for older adults (Chodzko-Zajko et al., 2009; Sherrington et al., 2011). The exercise training principles of progression and overload were applied in both interventions (Ammann et al., 2014), and were adapted to each participant's abilities to achieve a moderate to vigorous training intensity (Chodzko-Zajko et al., 2009). Twenty-four training sessions were performed within 8 weeks, with some participants missing certain sessions due to personal reasons.

**Figure 1:**
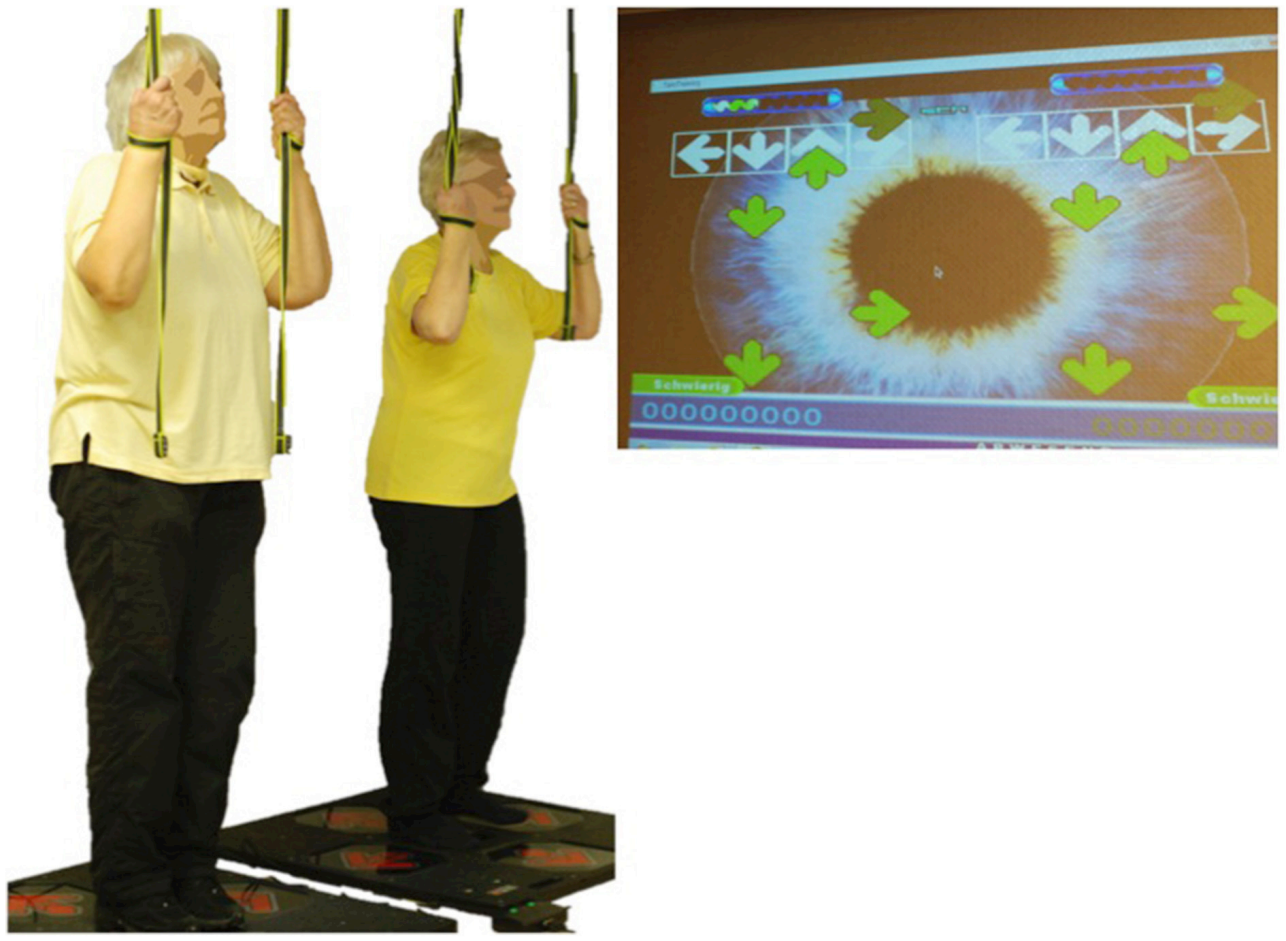
**Interactive cognitive-motor video game dancing (DANCE)**. Two participants perform steps on a pressure sensitive platform to the rhythm of the music. Step timing and direction are cued with arrows on a screen.

Intervention DANCE included interactive video game dancing, a so-called exergame, as a simultaneous cognitive-motor training (Figure 1). This training combines an attention demanding cognitive task with a simultaneous motor coordination aspect. Two Impact Dance Platforms (Positive Gaming BV, Haarlem, the Netherlands) were positioned side-by-side such that two participants could practice at the same time. Participants stood on the one-by-one meter platform, which contained four pressure sensitive areas to detect steps forward, backward, to the left, and to the right, respectively. Stepping sequences were cued with arrows appearing on a large screen. Steps had to be performed exactly when an arrow reached a highlighted area on the screen in order to achieve best scores in the game. Participants could hold on to ropes if necessary to maintain balance. Several levels of difficulty in stepping patterns and frequency were created with the StepMania software and different styles of music were chosen to add variety and meet participants' preferences. Training difficulty was adapted to each individual's coordination ability and was increased progressively.

Intervention BALANCE consisted of 20 min conventional balance training and 10 min stretching in each session. Balance training included two and single leg stand exercises, either on the floor or on various types of instable surfaces (e.g., foam and air pads, ropes, etc.). The stretching part of this intervention comprised four to five exercises for the major muscle groups. Stretching positions were held static for about 20–30 s.

### Assessment of prefrontal cortex activity during walking

Functional near-infrared spectroscopy (fNIRS) is a non-invasive optical imaging technique to measure blood flow changes in the brain associated with brain activity. Within the “optical window” from approximately 700–900 nm wavelength, light readily penetrates most biological tissues, including bone. Thereby oxygenated hemoglobin (HbO_2_) and deoxygenated hemoglobin (Hb) represent the most important light absorbers or chromophores within the optical window, besides water that remains constant (Ekkekakis, 2009). A multi distance, frequency domain fNIRS instrument (Oxiplex TS Tissue Spectrometer, ISS Inc., Champaign, IL, USA) was used to measure prefrontal cortex (PFC) activity during walking. This instrument emits a modulated light beam at two distinct wavelengths of 690 and 830 nm to measure absolute concentrations of HbO_2_ and Hb, respectively. The applied wavelengths have the advantage of low cross-talk (Boas et al., 2004). Moreover, multi distance technique, consisting of eight light emitting fibers placed at four different distances from the fiber optic detector, allowed to exclude measurement bias from skin blood flow changes during the walking experiment. Two of these sensors were tightly secured on the participants' forehead with a bandage to avoid displacement during walking and interference with extraneous light. The two sensors were placed according to the international 10/20 EEG electrode system, covering the areas between Fp1-F3-F7 and Fp2-F4-F8 that correspond to the left and the right PFC, respectively (Leff et al., 2008).

### fNIRS treadmill walking protocol

**Figure 2:**
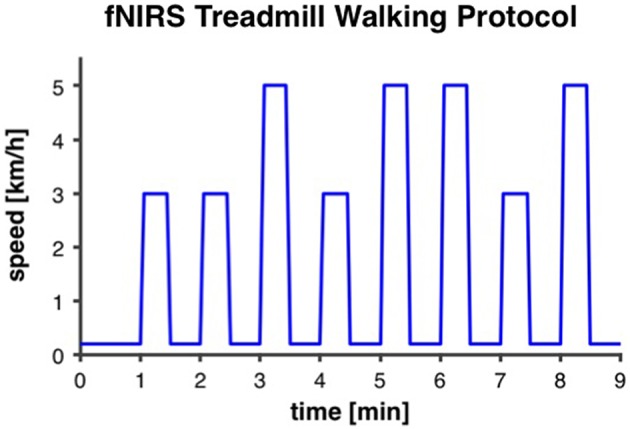
**Intermittent fNIRS treadmill walking protocol**. The protocol was repeated two times with 3 min recovery in between. It started with 1 min of very slow walking at 0.2 km/h for the measurement of baseline fNIRS-values. Thereafter, eight intervals of 30 s at either preferred or fast individual walking speed (3 and 5 km/h, respectively, in this example), with 30 s of rest at 0.2 km/h, were performed.

A similar treadmill protocol, as reported by Suzuki et al. (2004), was applied. Our intermittent walking protocol lasted for 9 min and was repeated two times with a recovery period of 3 min in between. Each 9-min trial started with 1 min of very slow walking at 0.2 km/h for the measurement of baseline fNIRS-values. Thereafter, eight intervals of 30 s at either preferred or fast individual walking speed (four intervals at each speed) were performed, with 30 s of rest at 0.2 km/h after each interval (Figure 2). In order to minimize anticipation effects, participants were not prepared or cued by the instructors about when the walking intervals would start and if the interval was going to be at preferred or fast walking speed. For that purpose the display of the treadmill was covered. Participants were allowed to hold on to the handrails of the treadmill and, if necessary due to insecure gait, were secured with a climbing harness that was attached to the ceiling to avoid falls. Before participants were equipped with the fNIRS sensors for the treadmill test, individual preferred walking speed was measured with a 4-Meter Walk test on the ground. Individual fast walking speed was then calculated by adding 2 km/h. This difference between preferred and fast walking speed was defined based on gait measurements in one of our previous studies with older adults (Eggenberger et al., 2015b). Thereafter, participants were accustomed to walking on the treadmill at different speeds for about 2–3 min to assure they were capable of walking at the two predefined speeds. For several participants, preferred walking speed as measured on the ground was subjectively rated as quite fast when performed on the treadmill and it was not possible for them to increase speed by 2 km/h. If this was the case, fast walking speed was reduced until it fitted the participant's abilities and preferred walking speed was set at 2 km/h below. The same individual walking speeds were applied for pre- and post-test measurements.

### Processing of fNIRS data

fNIRS data were recorded with a sampling frequency of 1 Hz and exported to Microsoft Excel for processing and analysis. Data from the two sensors on the left and right PFC, respectively, were analyzed separately. Raw data were de-trended and transformed to concentration change values (Δ μM) by subtracting a 60-s moving average as a high-pass filter. This procedure allowed for direct comparison of PFC activity between individuals and groups. After visual inspection of the variation range of the data, motion artifacts in HbO_2_-values were defined as >2.5 and < −2.5μM and were excluded from further analyses. Artifact cut-off for Hb-values was defined as >1.5 and < −1.5 μM. In five data sets with larger variations (two post-tests in DANCE, one pre-test and two post-tests in BALANCE), artifact cut-off range for HbO_2_ and Hb was extended by ±1.5 μM, respectively. From the two times 9 min treadmill walking protocol, 16 one-min data blocks comprising 30 s preferred or fast walking intervals, followed by 30 s rest were available. Time-triggered averages were calculated for the eight 1-min walk/rest blocks containing preferred walking intervals and the eight blocks with fast walking intervals. This procedure was applied to minimize bias from Mayer waves that change HbO_2_ concentrations with about 0.1 Hz frequency and about 0.1 μM amplitude (Julien, 2006). From the two 1-min phases, that preceded the first walking interval of each 9-min trial, a baseline average value was calculated from the 10th to the 50th second. After visual inspection of the time courses of HbO_2_ within the 1-min walk/rest blocks in the pre-test data, four timeframes were defined and averaged for further comparisons. The first timeframe was set at the initiation and acceleration of walking from the first to the seventh s of walking (t1–7), the second timeframe t10–25 represented “steady-state” walking, while the third timeframe t26–34 reflected the end of walking with deceleration and a drop of HbO_2_ back to baseline. The fourth timeframe t35-46 was chosen to analyze the drop of HbO_2_ below baseline during the rest phases.

### Secondary assessments

#### Cognitive performance tasks

Cognitive performance in executive functions, general cognitive ability, and information processing speed was assessed with five “paper-and-pencil” tasks. According to Miyake et al.'s model of executive functions (Miyake et al., 2000), these can be divided into three lower-level factors, which comprise shifting, inhibition, and working memory updating. Shifting was measured with the Trail Making Test part B (TMT-B; Lezak et al., 2012), inhibition was assessed with the Stroop Word-Color Interference task (Oswald and Fleischmann, 1997), and working memory was assessed with the Executive Control task (Baller et al., 2006). Furthermore, general cognitive ability was recorded using the Montreal Cognitive Assessment (MoCA; Nasreddine et al., 2005) and information processing speed was measured with the Trail Making Test part A (TMT-A; Lezak et al., 2012).

### Short physical performance battery

The Short Physical Performance Battery (SPPB) assesses lower extremity functioning (Guralnik et al., 1994). This test battery comprises a balance test, a 4-Meter Walk test, and a 5 Chair-Rises test. Full test administration criteria are available at the National Institute on Aging website (Guralnik, 2013). The standard SPPB balance test was extended with two additional levels of difficulty to avoid ceiling effects. The first additional level included a 20-s single-leg stance. One point was added to the SPPB balance test score when 10 s were reached and another point when 20 s were completed. For the second additional level, a single-leg stance with eyes closed was required to maintain for as long as possible. One point was added to the balance score for every 5 s the position was held.

#### Fear of falling and depression

With the Falls Efficacy Scale International (FES-I) fear of falling was assessed (Yardley et al., 2005), while symptoms of depression were measured with the German version of the Geriatric depression scale (GDS; Gauggel and Birkner, 1999).

#### Training enjoyment

Training enjoyment was recorded at post-test applying the German eight-item version of the Physical Activity Enjoyment Scale (PACES; Mullen et al., 2011; Jekauc et al., 2013). The average score of the eight items was used for statistical analysis.

### Statistical analysis

Baseline group differences of demographic characteristics, cognitive and physical performance, FES-I and GDS data were analyzed with unpaired student's *t*-tests. Two-way repeated measures analyses of variance (ANOVA), with “intervention” as between-subject factor and “time,” or “hemisphere,” or “walking speed” as within-subject factors, were applied to analyze differences in PFC activity between pre- and post-test, or left and right hemisphere, or preferred and fast walking variables, respectively. Pearson's correlation was calculated to identify associations of PFC activity changes from pre- to post-test with cognitive performance changes from pre- to post-test. A paired student's *t*-test was used to assess differences between baseline and experimental PFC activity levels at the fNIRS pre-test. Statistical calculations were performed with IBM SPSS Statistics software for Macintosh, version 22.0 (IBM Corp., Armonk, NY, USA). A significance level of α = 0.05 was applied and effect size *r* was defined as small at *r* = 0.10, medium at *r* = 0.30, and large at *r* = 0.50 and above (Cohen, 1988).

## Results

**Figure 3:**
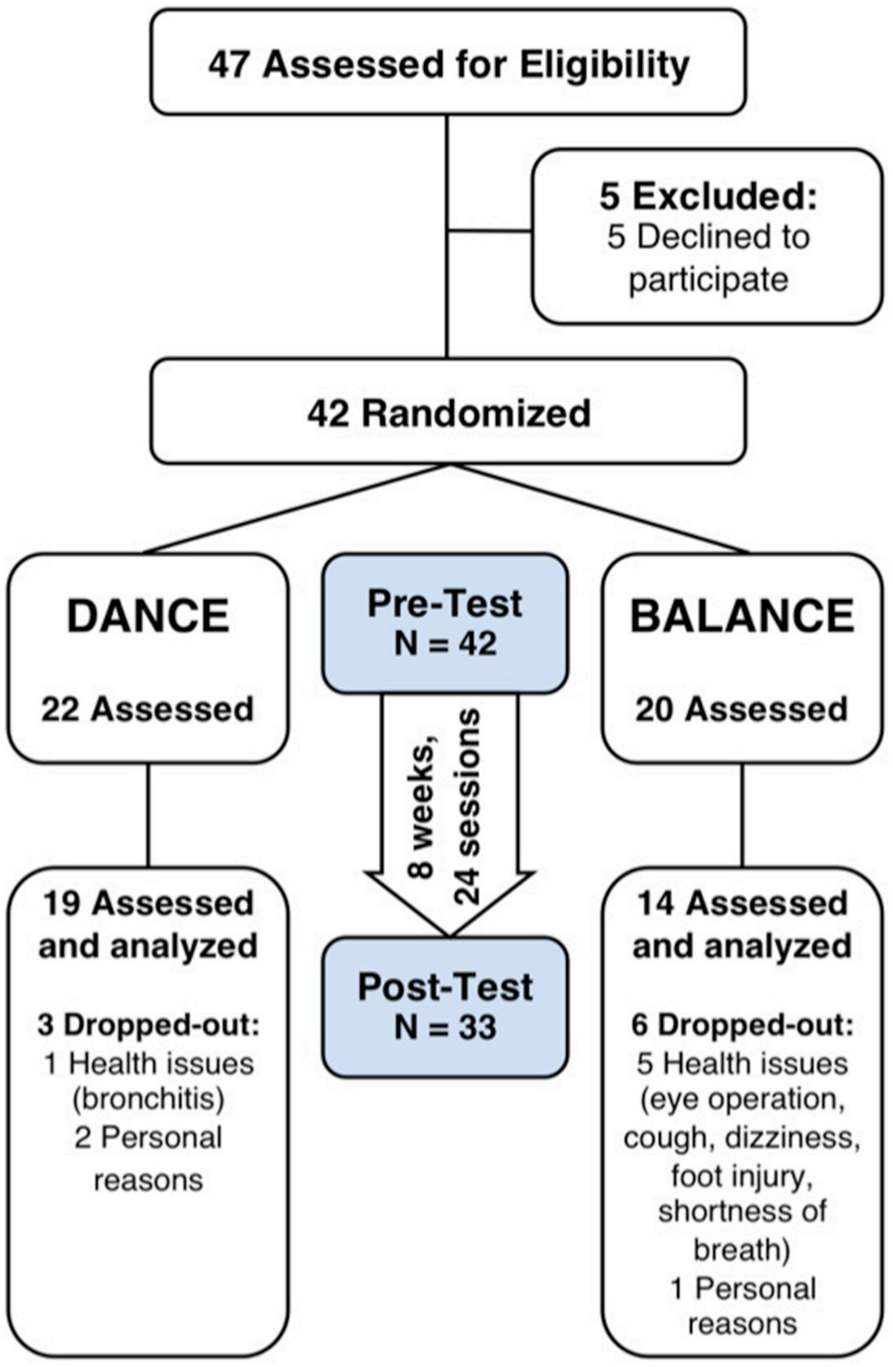
**Participants' flow and trial design**. Participants were randomly assigned to either DANCE or BALANCE and were trained over 8 weeks three times per week for 30 min. fNIRS prefrontal cortex activity during treadmill walking, cognitive performance, Short Physical Performance Battery, fear of falling, and depression were assessed at pre- and post-test. DANCE, video game dance training; BALANCE, balance and stretching training.

Thirty-three participants completed the 8-week exercise training intervention (21.4% attrition). Participants' flow is presented in Figure 3. We did not apply strict intention-to-treat analysis in favor of a clear declaration of the reasons for dropouts, as recommended by the CONSORT 2010 guidelines (Moher et al., 2010). Dropouts due to health issues were not associated to the intervention and dropouts due to personal reasons were equally distributed between groups. Therefore, nine participants who did not complete the trial were excluded from pre-/post-test analyses. Table 1 shows demographics and training characteristics (enjoyment, intensity, and compliance) of the two intervention groups.

**Table 1:** **Demographics and training characteristics**.

**Variable**	**DANCE**	**BALANCE**	***p*-value, two-tailed**
*N*	19	14	
Gender, female	12, 63.2%	9, 64.3%	
Age, years	72.8 (5.9)	77.8 (7.4)	0.039^*^
Height, cm	169.4 (9.3)	167.1 (8.8)	0.477
Weight, kg	70.0 (14.9)	64.9 (11.5)	0.290
BMI, kg/m^2^	24.4 (5.0)	23.1 (2.9)	0.402
Education, years	13.4 (1.8)	13.6 (2.1)	0.830
Training compliance (24 sessions)	91.4% (7.1%)	94.3% (6.2%)	0.234
Training enjoyment, PACES score (1–7)	6.13 (0.76)	6.11 (0.82)	0.931
Training intensity perceived physically, RPE (1–10)	5.0 (2.0)	5.5 (2.1)	0.515
Training intensity perceived cognitively, RPE (1–10)	5.4 (2.0)	4.6 (2.4)	0.314
Preferred treadmill walking speed, km/h	3.8 (0.6)	3.7 (0.6)	0.818
Fast treadmill walking speed, km/h	5.8 (0.6)	5.7 (0.6)	0.818

Data are numbers or means (± standard deviation in brackets). Bold values indicate significance,

* p < 0.05;

BMI, Body Mass Index; PACES, Physical Activity Enjoyment Scale; RPE, rate of perceived exertion; DANCE, video game dance training; BALANCE, balance and stretching training.

### fNIRS treadmill test

#### Comparison of pre- vs. post-test PFC activity

**Figure 4:**
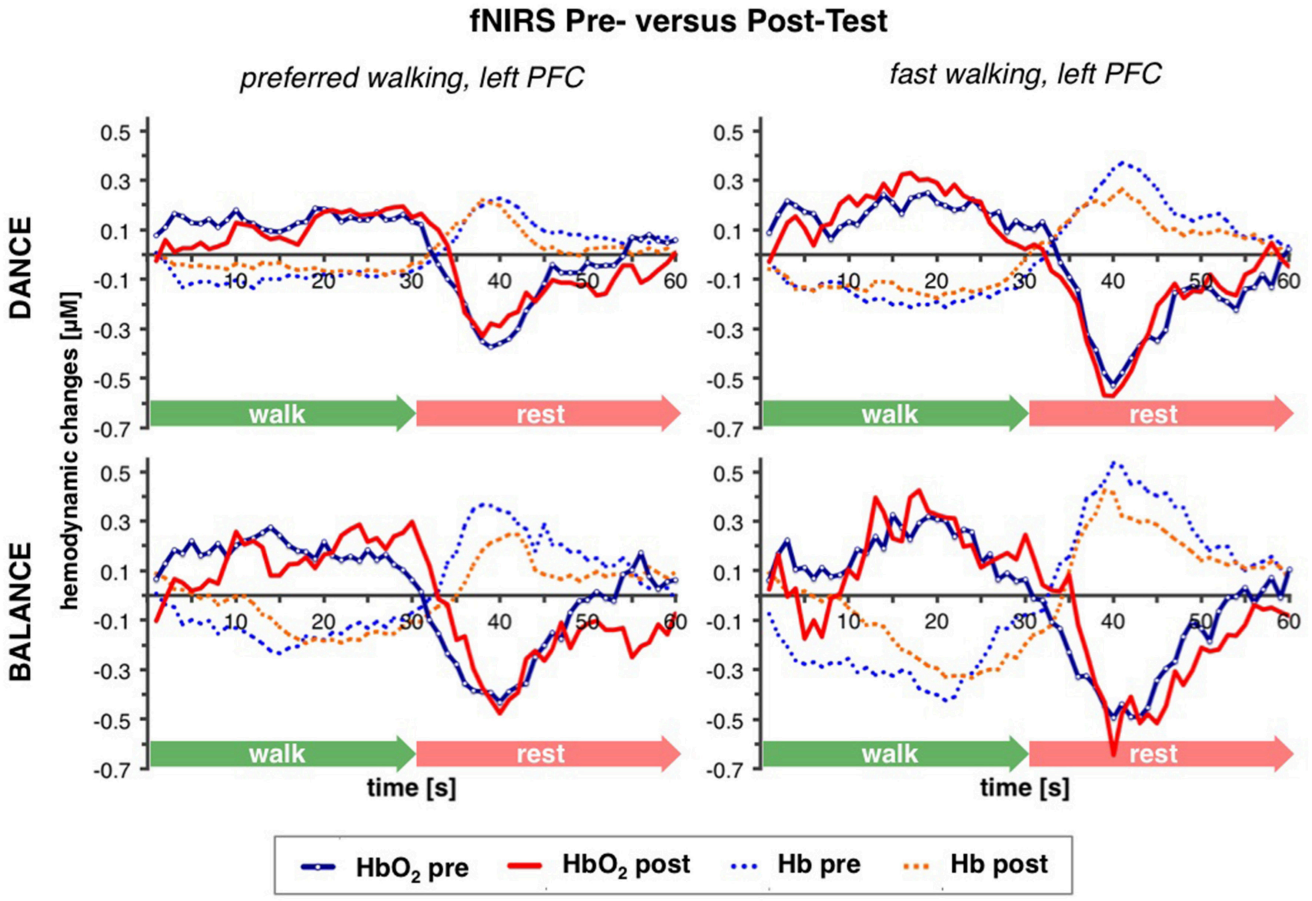
**Comparison of pre- vs. post-test PFC activity**. Graphs represent time-triggered group averages. Identical line color and style in Figures 4–**6** represent the same data. Within the first 7 s of walking (t1–7) HbO_2_ concentration was significantly reduced at post-test at preferred walking speed (graphs on the left side), whereas a trend for a reduced activity was evident at fast walking speed in the same timeframe (graphs on the right side). At the end of fast walking (t26–34, graphs on the right side) a significant time × intervention interaction reflected reduced HbO_2_ concentration in DANCE and increased HbO_2_ in BALANCE. DANCE, video game dance training; BALANCE, balance and stretching training, PFC, prefrontal cortex.

Table 2 depicts statistical results of pre- vs. post-test PFC activity within three timeframes during preferred or fast walking, and in the left and right hemisphere. Figure 4 shows the time-triggered group averages for the left PFC fNIRS sensor. HbO_2_ concentration was significantly reduced at post-test within t1–7 at preferred walking speed in the left and right PFC, whereas a trend for a reduced activity was evident at fast walking speed in the left PFC. Additionally, during t26–34 a significant time × intervention interaction reflected reduced HbO_2_ concentration in DANCE and increased HbO_2_ in BALANCE.

**Table 2:** **Results of repeated measures ANOVA for pre- vs. post-test PFC activity**.

**HbO**_**2**_ **variables**			**Main effect (time, pre vs. post)**				**Interaction effect (time × intervention)**		
**Averaged time-frame**	**Walking speed**	**Hemisphere**	***df***	***F***	***p*, one-tailed**	***r***	***F***	***p*, one-tailed**	***r***
t1–7	Preferred	Left PFC	(1, 31)	4.59	0.020^*^	**0.36**	0.16	0.344	0.07
		Right PFC	(1, 29)	3.32	0.040^*^	**0.32**	1.54	0.112	0.22
	Fast	Left PFC	(1, 31)	2.03	**0.082^t^**	**0.25**	0.28	0.300	0.09
		Right PFC	(1, 29)	0.60	0.224	0.14	0.03	0.435	0.03
t10–25	Preferred	Left PFC	(1, 31)	0.25	0.311	0.09	0.02	0.450	0.02
		Right PFC	(1, 29)	0.46	0.251	0.13	0.13	0.360	0.07
	Fast	Left PFC	(1, 31)	0.48	0.247	0.12	0.04	0.422	0.04
		Right PFC	(1, 29)	0.18	0.337	0.08	0.09	0.385	0.05
t26–34	Preferred	Left PFC	(1, 31)	5.52	(0.013)	0.39	1.32	0.130	0.20
		Right PFC	(1, 29)	0.68	0.208	0.15	0.76	0.196	0.16
	Fast	Left PFC	(1, 31)	0.08	0.393	0.05	3.54	0.035^*^	**0.32**
		Right PFC	(1, 29)	0.23	0.318	0.09	0.04	0.418	0.04

Bold values indicate trend or significance,

t p < 0.10 trend,

* p < 0.05, significant p-value in brackets does not correspond with one-sided hypothesis; PFC, prefrontal cortex.

#### Comparison of left vs. right PFC activity

**Figure 5:**
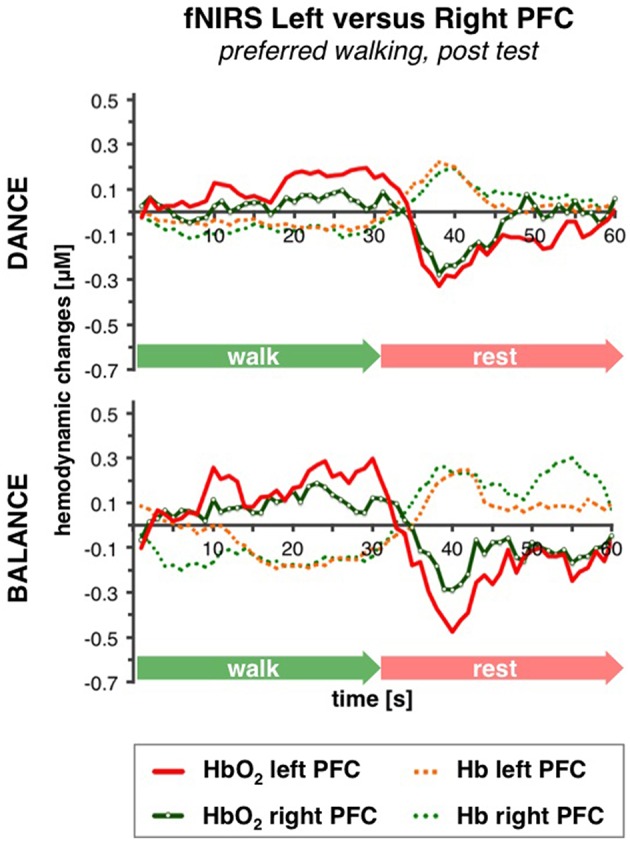
**Comparison of left vs. right PFC activity**. Graphs represent time-triggered group averages. Identical line color and style in Figures 4–**6** represent the same data. Within the timeframes t10–25 and t26–34 left PFC activity was higher than right PFC activity in DANCE and BALANCE. No differences in left vs. right PFC activity during walking were present at pre-test (graphs not shown). DANCE, video game dance training; BALANCE, balance and stretching training, PFC, prefrontal cortex.

No differences in left vs. right PFC activity during walking were present at pre-test (statistical data are presented in Table 3). At post-test, within t10–25 and t26–34, at preferred and fast walking speed, left PFC activity was higher than right PFC activity. Two trends for significant hemisphere × intervention interaction effects were found at fast walking in t1–7 and t26–34, which represent equal left vs. right PFC activity in DANCE, and lower (t1–7) or higher (t26–34) left compared to right PFC activity in BALANCE. Figure 5 shows time triggered group averages at preferred walking for left vs. right PFC at post-test.

**Table 3:** **Results of repeated measures ANOVA for left vs. right PFC activity**.

**HbO**_**2**_ **variables**			**Main effect (hemisphere, left vs. right)**				**Interaction effect (hemisphere× intervention)**		
**Averaged time-frame**	**Walking speed**	**Test time**	***df***	***F***	***p*, one-tailed**	***r***	***F***	***p*, one-tailed**	***r***
t1–7	Preferred	Pre	(1, 29)	0.06	0.403	0.05	0.36	0.276	0.11
		Post	(1, 31)	0.01	0.456	0.02	0.15	0.353	0.07
	Fast	Pre	(1, 29)	1.00	0.163	0.18	0.33	0.285	0.11
		Post	(1, 31)	1.61	0.107	0.22	1.74	**0.099^t^**	**0.23**
t10-25	Preferred	Pre	(1, 29)	0.80	0.190	0.16	0.57	0.229	0.14
		Post	(1, 31)	2.34	**0.069^t^**	**0.26**	0.02	0.444	0.03
	Fast	Pre	(1, 29)	1.05	0.158	0.19	0.25	0.311	0.09
		Post	(1, 31)	3.42	0.037^*^	**0.32**	0.01	0.454	0.02
t26-34	Preferred	Pre	(1, 29)	0.01	0.457	0.02	0.06	0.403	0.05
		Post	(1, 31)	3.58	0.034^*^	**0.32**	0.07	0.398	0.05
	Fast	Pre	(1, 29)	0.02	0.451	0.02	0.30	0.293	0.10
		Post	(1, 31)	2.96	0.048^*^	**0.30**	2.53	**0.061^t^**	**0.27**

Bold values indicate trend or significance,

t p < 0.10 trend,

* p < 0.05; PFC, prefrontal cortex.

#### Comparison of preferred vs. fast walking PFC activity

**Figure 6:**
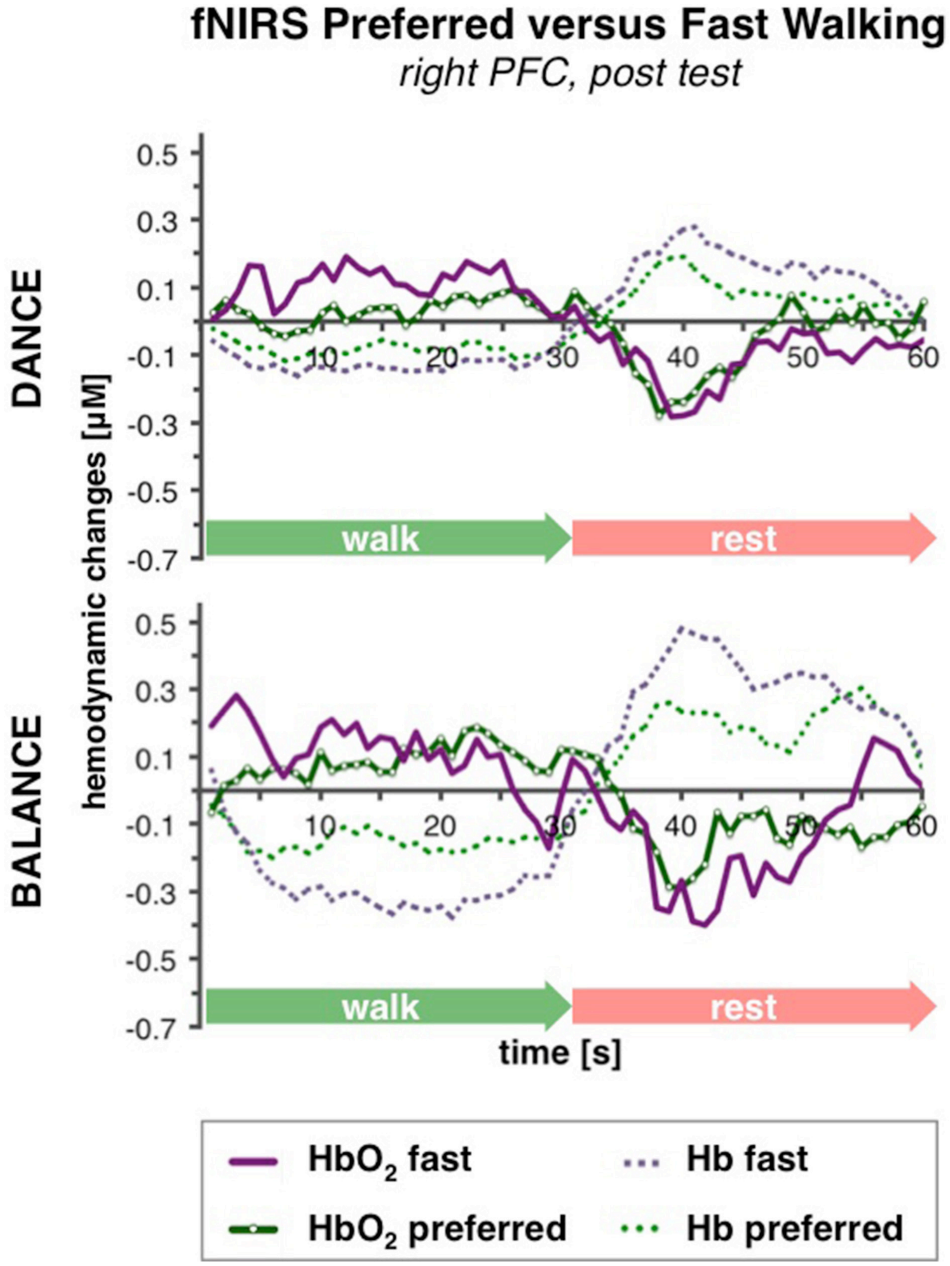
**Comparison of preferred vs. fast walking PFC activity**. Graphs represent time-triggered group averages. Identical line color and style in Figures 4–6 represent the same data. Within the timeframes t1–7 and t10–25 HbO_2_ levels were higher at fast compared to preferred walking speed in DANCE and BALANCE. PFC activity at pre-test was equal for preferred and fast walking speed (graphs not shown). DANCE, video game dance training; BALANCE, balance and stretching training, PFC, prefrontal cortex.

PFC activity at pre-test was equal for preferred and fast walking speed (for statistical data see Table 4). At post-test, HbO_2_ levels were higher at fast compared to preferred walking speed within t1–7 in the right PFC, and within t10–25 in the left and right PFC. Time triggered group averages in the right PFC for preferred vs. fast walking at post-test are presented in Figure 6.

**Table 4:** **Results of repeated measures ANOVA for preferred vs. fast walking speed PFC activity**.

**HbO**_**2**_ **variables**			**Main effect (walking speed, preferred vs. fast)**				**Interaction effect (speed × intervention)**		
**Averaged time-frame**	**Hemisphere**	**Test-time**	***df***	***F***	***p*, one-tailed**	***r***	***F***	***p*, one-tailed**	***r***
t1–7	Left PFC	Pre	(1, 31)	0.00	0.476	0.01	0.53	0.236	0.13
		Post	(1, 31)	0.01	0.464	0.02	1.39	0.124	0.21
	Right PFC	Pre	(1, 29)	0.56	0.231	0.14	0.15	0.352	0.07
		Post	(1, 31)	3.00	0.047^*^	**0.30**	0.27	0.305	0.09
t10–25	Left PFC	Pre	(1, 31)	1.41	0.123	0.21	0.04	0.424	0.03
		Post	(1, 31)	6.22	.009^**^	**0.41**	0.32	0.288	0.10
	Right PFC	Pre	(1, 29)	0.10	0.379	0.06	0.09	0.385	0.05
		Post	(1, 31)	1.93	**0.088**^*t*^	**0.24**	0.84	0.184	0.16
t26–34	Left PFC	Pre	(1, 31)	0.10	0.376	0.06	0.04	0.418	0.04
		Post	(1, 31)	2.17	(0.075)	0.26	0.83	0.185	0.16
	Right PFC	Pre	(1, 29)	0.00	0.492	0.00	0.58	0.226	0.14
		Post	(1, 31)	2.28	(0.071)	0.26	1.13	0.149	0.19

Bold values indicate trend or significance,

t p < 0.10 trend,

* p < 0.05,

** p < 0.01, significant p-values in brackets do not correspond with one-sided hypothesis; PFC, prefrontal cortex.

### Correlation of pre-/post-test changes in PFC activity and cognitive performance

Table 5 shows correlation coefficients for pre- to post-test changes in PFC activity and cognitive performance. Reduced time (improvement) to complete TMT-A and TMT-B correlated with reduced HbO_2_ activity in the left PFC at preferred and fast walking in t1–7. Increased performance in TMT-A also correlated with reduced right PFC activity at preferred walking in t10–25 and at fast walking in t26–34. A reduction in Stroop word-color interference time (improvement) correlated with elevated right PFC activity at fast walking in t1–7, while reduced activity in this HbO_2_ variable was associated with increased performance in the Executive Control task. Additionally, an improvement in the Executive Control task correlated also with a reduction in right PFC activity at fast walking in t10-25.

**Table 5:** **Results of Pearson's correlation analysis between pre-/post-test changes in cognitive performance and PFC activity**.

**HbO**_**2**_ **variables**			**Cognitive performance variables**							
			**TMT-B (EF, shifting)**		**Stroop word-color (EF, inhibition)**		**Executive control (EF, working) memory)**		**TMT-A (processing) speed)**	
**Averaged time-frame**	**Walking speed**	**Hemisphere**	***r***	***p*, two-tailed**	***r***	***p*, two-tailed**	***r***	***p*, two-tailed**	***r***	***p*, two-tailed**
t1–7	Preferred	Left PFC	**0.34**	**0.051**^t^	0.16	0.366	−0.08	0.639	**0.33**	**0.062**^t^
		Right PFC	−0.05	0.789	0.23	0.208	−0.29	0.119	0.04	0.843
	Fast	Left PFC	**0.39**	0.023^*^	−0.15	0.391	−0.06	0.759	**0.37**	0.036^*^
		Right PFC	−0.03	0.879	−**0.43**	0.016^*^	−**0.33**	**0.069**^t^	−0.04	0.814
t10-25	Preferred	Left PFC	0.13	0.465	−0.09	0.616	0.08	0.673	0.03	0.877
		Right PFC	−0.03	0.891	−0.15	0.417	−0.18	0.337	**0.31**	**0.089**^t^
	Fast	Left PFC	0.11	0.560	−0.12	0.517	−0.23	0.207	−0.17	0.356
		Right PFC	0.01	0.970	0.13	0.494	−**0.35**	**0.056**^t^	0.05	0.788
t26-34	Preferred	Left PFC	−0.13	0.474	−0.12	0.502	0.09	0.636	−0.18	0.325
		Right PFC	−0.05	0.788	−0.12	0.507	0.11	0.547	0.00	0.996
	Fast	Left PFC	−0.01	0.943	−0.19	0.296	0.02	0.908	−0.03	0.883
		Right PFC	0.00	0.988	0.12	0.522	−0.06	0.753	**0.50**	0.004^**^

Bold values indicate trend or significance,

t p < 0.10 trend,

* p < 0.05,

** p < 0.01; EF, executive function; TMT-A, Trailmaking Test A; TMT-B, Trailmaking Test B; PFC, prefrontal cortex.

#### Comparison of baseline vs. experimental PFC activity at pre-test

All 42 participants who performed the fNIRS pre-test session where included in this comparison of HbO_2_ baseline vs. experimental values. Differences between the baseline HbO_2_-values and the experimental walking or rest interval HbO_2_-values were evident in almost all variables, except in t26-34 at fast walking as shown in detail in Table 6.

**Table 6:** **Results of paired t-test for baseline vs. experimental PFC activity at pre-test**.

**Experimental HbO**_**2**_ **variables**			**Paired** ***t*****-test**			
**Averaged Time-frame**	**Walking speed**	**Hemisphere**	***df***	***t***	***p*, one-tailed**	***r***
t1–7	Preferred	Left PFC	(1, 40)	−2.27	0.014^*^	**0.34**
		Right PFC	(1, 38)	−1.98	0.027^*^	**0.31**
	Fast	Left PFC	(1, 40)	−1.89	0.033^*^	**0.29**
		Right PFC	(1, 38)	−2.48	0.009^**^	**0.37**
t10–25	Preferred	Left PFC	(1, 40)	−3.20	0.001^**^	**0.45**
		Right PFC	(1, 38)	−1.92	0.031^*^	**0.30**
	Fast	Left PFC	(1, 40)	−3.26	0.001^**^	**0.46**
		Right PFC	(1, 38)	−1.95	0.029^*^	**0.30**
t26–34	Preferred	Left PFC	(1, 40)	−1.58	**0.061**^t^	**0.24**
		Right PFC	(1, 38)	−1.98	0.027^*^	**0.31**
	Fast	Left PFC	(1, 40)	−0.85	0.200	0.13
		Right PFC	(1, 38)	−0.52	0.302	0.08
t35–46	Preferred	left PFC	(1, 40)	4.77	<0.001^***^	**0.60**
		Right PFC	(1, 38)	2.52	0.008^**^	**0.38**
	Fast	Left PFC	(1, 40)	5.17	<0.001^***^	**0.63**
		Right PFC	(1, 38)	2.59	0.007^**^	**0.39**

Bold values indicate trend or significance,

t p < 0.10 trend,

* *p < 0.05,

** p < 0.01,

*** p < 0.001; PFC, prefrontal cortex.

### Secondary assessments

Baseline cognitive performance showed a significant group difference in the Stroop Word-Color Interference task (*p* = 0.035), whereas no baseline differences were present in the other tasks (TMT B *p* = 0.394, Executive Control *p* = 0.630, MoCA *p* = 0.383, TMT A *p* = 0.386). Baseline physical performance was not different between groups (SPPB total score *p* = 0.678, 4-Meter Walk *p* = 0.839, 5 Chair-Rises *p* = 0.740, Extended Balance test *p* = 0.811). Baseline values showed a trend to a significant difference between groups in FES-I (*p* = 0.098 trend) and no significant difference in GDS (*p* = 0.104). Performance development and statistical data of two-way repeated measures ANOVA from pre- to post-test are presented in Table 7.

**Table 7:** **Performance and statistical data of secondary assessments**.

**Variable**		**DANCE**		**BALANCE**		**ANOVA**			
	**Time**	**Mean**	***SE***	**Mean**	***SE***	**Effect**	***F*_(1, 31)_**	***p*, two-tailed**	***r***
**EXECUTIVE FUNCTIONS**									
Trail making B (s)	Pre	90.4	7.4	99.6	7.1	^*t*^	12.27	0.001^**^	**0.53**
	Post	74.9	6.5	79.6	5.6	*t* × *I*	0.19	0.665	0.08
Executive control (items)	Pre	9.79	1.29	8.93	1.09	*t*	1.88	0.180	0.24
	Post	10.84	1.10	10.57	0.88	*t* × *I*	0.09	0.766	0.05
Stroop word-color (s)	Pre	41.6	2.0	49.6	3.2	*t*	28.18	<0.001^***^	**0.69**
	Post	39.6	2.0	42.1	2.5	*t* × *I*	9.52	0.004^**^	**0.48**
**GENERAL COGNITION**									
MoCA (score)	Pre	25.95	0.58	26.64	0.48	*t*	9.43	0.004^**^	**0.48**
	Post	27.26	0.59	27.71	0.45	*t* × *I*	0.10	0.755	0.06
**PROCESSING SPEED**									
Trail making A (s)	Pre	39.8	3.5	44.3	3.6	*t*	10.34	0.003^**^	**0.50**
	Post	34.3	2.6	38.5	3.0	*t* × *I*	0.01	0.927	0.02
**PHYSICAL FUNCTIONING**									
SPPB (score)	Pre	11.58	0.16	11.43	0.36	*t*	0.79	0.382	0.16
	Post	11.47	0.30	11.86	0.14	*t* × *I*	2.14	0.153	0.25
4-Meter walk (s)	Pre	3.4	0.1	3.5	0.1	*t*	0.03	0.858	0.03
	Post	3.4	0.1	3.5	0.2	*t* × *I*	0.02	0.892	0.02
5 Chair-rises (s)	Pre	9.0	0.5	9.3	0.6	*t*	10.51	0.003^**^	**0.50**
	Post	7.8	0.4	8.3	0.5	*t* × *I*	0.16	0.695	0.07
Extended balance (score)	Pre	4.95	0.31	5.07	0.43	*t*	2.84	0.102	0.29
	Post	4.95	0.42	5.79	0.33	*t* × *I*	2.84	0.102	0.29
**FEAR OF FALLING AND DEPRESSION**									
FES-I (score)	Pre	20.84	1.21	18.57	0.54	*t*	2.97	**0.095**^*t*^	**0.30**
	Post	19.05	0.53	18.21	0.38	*t* × *I*	1.32	0.259	0.20
GDS (score)	Pre	4.47	0.76	2.86	0.59	*t*	0.00	0.979	0.01
	Post	4.53	0.78	2.79	0.55	*t* × *I*	0.03	0.864	0.03

Bold values indicate trend or significance,

t p < 0.10 trend,

*p < 0.05,

** p < 0.01,

*** p < 0.001;

t, time; t × I, time × intervention interaction; SE, standard error of measurement; SPPB, Short Physical Performance Battery; MoCA, Montreal Cognitive Assessment; FES-I, Falls Efficacy Scale International; GDS, Geriatric Depression Scale; DANCE, video game dance training; BALANCE, balance and stretching training.

## Discussion

In this study, we investigated the impact of two different exercise training interventions on fNIRS brain activity during walking and on executive functions in older adults. Thereby, one intervention combined cognitive and motor training simultaneously in an exergame (interactive video game dancing, DANCE), whereas the other intervention consisted of exclusive motor training (balance and stretching exercises, BALANCE). Our results demonstrated (1) that both interventions reduced left and right hemispheric PFC oxygenation during the acceleration of walking, while DANCE showed a larger reduction at the end of the walking compared to BALANCE in the left PFC, and (2) that the exercise training induced modulations in PFC oxygenation were associated with improved executive functions. The present study provides novel and important insight into the relation and mediation between exercise training, brain function during walking, and cognition in older adults.

### Reduced prefrontal brain activity during walking after exercise training

The fNIRS treadmill walking experiment supports our first hypothesis that 8 weeks of DANCE or BALANCE training induce a reduction of prefrontal brain activity during walking. This was particularly evident in the acceleration phase during the first 7 s of walking in both hemispheres and in both intervention groups. To date, no other study that we are aware of has longitudinally investigated exercise training related alterations of PFC activity during walking. Nevertheless, our results convene with previous findings from cognitive training studies. For instance, Erickson et al. (2007b) demonstrated reduced activity in several brain regions after cognitive dual-task training in older adults. Additionally, in our study, DANCE showed an earlier reduction of left PFC activity at the end of the 30 s walking interval after the training intervention. This observation is comparable to a cross-sectional fNIRS study that reported an earlier decrease of PFC activity already during the performance of a cognitive-motor dual-task in young adults, whereas PFC activity in the older adults remained elevated about 10 s beyond the end of the task (Ohsugi et al., 2013). Thereby, brain activity in the old adults resembles the untrained state of our participants at pre-test, whereas brain activity in the young adults is similar to our post-test results in DANCE.

Traditionally balance training is another option to train motor skills in older adults and effect on brain properties and cognitive performance (Voelcker-Rehage et al., 2011). Training programs that contain balance components counteract risk factors for falls and restore balance control in older adults. However, the positive effects cannot be exclusively attributed to balance exercise, because all the studies taken into account used a combination of strength and balance training (Taube et al., 2008). Moreover, the authors of this review concluded that the brain adaptions to balance training are rather task-specific and hitherto little is known about the influence of balance training influencing brain plasticity. The findings of our study warrant further investigations about the relation between conventional types of balance training and the effects on brain functioning during functional movement tasks.

Generally, decreased activity of a particular brain area may represent decreased use and, therefore, increased efficiency according to the reviews by Lustig et al. (2009) and Grady (2012). Additional brain activity in older compared to younger adults was suggested to reflect a compensatory mechanism (*compensation hypothesis*; Cabeza et al., 2002; Reuter-Lorenz and Cappell, 2008) to improve performance in a specific task and such over-recruitment might as well be associated with less efficient use of neural resources (Grady, 2012). This relation has not only been observed between young and old adults but also between higher fit and lower fit old adults. For instance, Voelcker-Rehage et al. (2010) reported that both high physical and high motor fitness were associated with reduced fMRI signals in frontal and other brain regions during cognitive tasks, and Harada et al. (2009) found that frontal brain activity in fast walking was higher in individuals with lower gait capacity as assessed with fNIRS. Additionally, decreased activity of prefrontal areas could also have resulted from a shift from controlled gait to more automatic gait or a shift from the indirect to the direct locomotor pathway, respectively (Hamacher et al., 2015). While the indirect locomotor pathway regulates gait via prefrontal cortex, premotor area, supplementary motor area, and basal ganglia, the direct pathway comprises primary motor cortex (M1), cerebellum, and spinal cord (la Fougere et al., 2010; Zwergal et al., 2012). Similar observations of reduced brain activity have also been reported in studies with highly skilled, professional pianists (Jancke et al., 2000) or soccer players (Naito and Hirose, 2014) that both showed lower recruitment of motor areas in which they have richer sensory-motor experiences (finger or foot movements, respectively) compared to amateurs. The authors explained their findings with highly efficient motor control processes due to over-years training of a motor skill, which led to a smaller amount of neurons needed to perform the particular skill. Together, the effects in both interventions and particularly in DANCE might reflect an adaptation toward the function of a younger or more trained brain.

### Hemispheric asymmetry in prefrontal brain activity after exercise training

The comparison of brain activity in the left vs. the right PFC showed that hemispheric differences only became obvious at the end of both 8-week training interventions at post-test. At this time point of measurement, left PFC activity was larger than right PFC activity at preferred and fast walking speed during the “steady state” walking phase (t10–25) and also at the end of the walking interval (t26–34). To the best of our knowledge and according to the review by Ekkekakis (2009), prefrontal hemispheric asymmetry has not yet been investigated with fNIRS in relation to physical exercise training. Nonetheless, our observation is in line with a cognitive training fMRI study that found increased post-test prefrontal hemispheric asymmetry during dual-task conditions (Erickson et al., 2007b). In particular, the authors reported an elevation of left ventrolateral PFC activity and a reduction of right ventrolateral PFC activity in the old adults after training and a concomitant reduction in age differences compared to young adults. Similarly, Bergerbest et al. (2009) demonstrated in their fMRI study on implicit memory (repetition priming) an initial bilateral PFC activity in older adults and left lateralized activity in younger adults. These initial activity patterns were followed by repetition related activity reductions, which were smaller for the older compared to the younger adults in the left PFC but larger in the right PFC. They assumed that the initial right PFC activity in the elderly would compensate for a lower aging-related left hemispheric PFC activity. These and our own findings correspond with the *complementary hypothesis* (Colcombe et al., 2005), proposing that in older adults bilateral brain activity or a reduction of asymmetric brain activity is not generally related to better performance in a certain task, as it is suggested by the *compensation hypothesis* (Cabeza et al., 2002; Reuter-Lorenz and Cappell, 2008). Moreover, Lustig et al. (2009) hypothesized in their comprehensive review that compensatory effects seen in older adults, such as the aforementioned activation of additional contralateral brain regions for better performance, would typically occur in single-session studies, whereas after several sessions of training, brain activity patterns would become more similar to young adults. Based on this framework, our finding of exercise training induced increased hemispheric asymmetry during treadmill walking, could be explained with less need for compensation through right prefrontal activity and might represent another adaption toward the function of a younger adults' brain.

### Gait speed related differences in prefrontal brain activity after exercise training

Gait speed related differences in PFC activity were not found at pre-test but only at post-test, which converges with the previously discussed hemispheric differences. Post-test differences were evident in the right PFC during acceleration (t1–7) and in both PFC hemispheres during “steady state” walking (t10–25). Our pre-test result is consistent with a cross-sectional study in old adults (mean age 63 ± 4 years) that also did not find differences in PFC oxygenation related to gait speeds comparable with those in our study (50 and 30% intensity of individual heart rate response, respectively; Harada et al., 2009). Similarly, two fNIRS studies with young adults did not find changes in PFC activity when preferred walking speed was increased by either 20% (Meester et al., 2014) or from 3 to 5 km/h (Suzuki et al., 2004). However, the latter study found significantly elevated PFC activity when the young participants were running at 9 km/h, and in the aforementioned study by Harada et al. (2009), PFC activity increased in the older adults when they walked at 70% intensity. It can, therefore, be concluded that changes in PFC activity in young and old adults only occur when the difference in locomotion speed between conditions is large enough.

The question, however, remains what mechanism could have led to gait speed related differences in prefrontal oxygenation at post-test in our study? Several cross-sectional studies with young and old adults compared brain activity of normal vs. challenging walking tasks, including different dual-task walking conditions, walking in dim lighting, negotiating obstacles, etc. (Holtzer et al., 2011; Clark et al., 2014; Meester et al., 2014; Mirelman et al., 2014). They consistently demonstrated increased PFC activity during challenging walking compared to normal walking. Noteworthy, goal-directed locomotion, such as more complex walking tasks and dual-task walking, are associated with increased activation of the indirect locomotor pathway via prefrontal areas (Hamacher et al., 2015). Interestingly, it was found that PFC activity in complex walking tasks was elevated more in older adults with better gait quality (Clark et al., 2014) or in young adults compared to old adults (Holtzer et al., 2011), referring to *under-recruitment* in the older or less fit old adults, respectively. In correspondence with our study these findings may reflect the transition from an untrained to a trained state. In contrast, the study by Harada et al. (2009) observed that PFC activity during fast walking (at 70% intensity) was elevated in the elderly with lower gait capacity which refers to *over-recruitment*. At first sight this looks like a discrepancy, however, might be explained by recent findings from Kennedy et al. (2015) and earlier studies (Mattay et al., 2006; Reuter-Lorenz and Cappell, 2008). These authors demonstrated that compensatory brain activity (over-recruitment) is effective in relation to lower task difficulty, while with increasing task complexity older adults reach a resource ceiling leading to less activity compared to the younger adults (under-recruitment). Moreover, Clark et al. (2014) and Holtzer et al. (2011) proposed that the ability to increase PFC activity in challenging walking situations would be an important mechanism to optimize performance.

These conceptions are in line with the *frontal lobe hypothesis of aging* (West, 1996) and with the *cognitive reserve theory* which assumes that younger adults increase brain activity by a larger degree to cope with elevated cognitive task difficulty (Stern, 2009). Notably, the age-related neural modulation patterns of functional over- and under-recruitment are emerging especially within the transition from middle-aged to old adults (Kennedy et al., 2015). Moreover, these functional effects of aging are mirroring age-related structural effects proposed by the “*last-in-first-out” hypothesis* where late maturing brain regions decline first in later life (Raz and Kennedy, 2009; Tamnes et al., 2013; Bender et al., 2015) and explain gait disturbances (Scherder et al., 2011). We conclude, therefore, that in our study the ability to differentiate PFC activity related to walking speed was either attenuated due to reduced availability or underutilization of PFC resources at pre-test when the older participants were in an untrained state. The walking speed related differences in PFC activity that we found at post-test may, therefore, again reflect a mechanism of how the older adults' brain adapted its function toward a young adult-like brain.

### Correlated changes in prefrontal brain activity and cognitive performance

Correlation analysis is supporting our second hypothesis and revealed associated exercise training induced changes in prefrontal brain activity and improvements in different behavioral measures of executive function and processing speed. This association could be explained by the fact that the PFC represents a brain area that is involved in both locomotion (Suzuki et al., 2004) and in cognitive executive functioning (Shibuya-Tayoshi et al., 2007). The results of our correlation analysis extend recent findings from several exercise training studies that employed fMRI to assess brain functional or structural adaptations alongside with cognitive performance measures (Voss et al., 2010, 2013; Erickson et al., 2011; Voelcker-Rehage et al., 2011; Liu-Ambrose et al., 2012; ten Brinke et al., 2015). For instance, Voelcker-Rehage et al. (2011) found training-specific brain functional adaptations during the performance of an executive function task (flanker task) after both aerobic and coordination training. These adaptations were accompanied by increased cognitive executive performance. Similarly, Liu-Ambrose et al. (2012) reported correlating brain functional and cognitive plasticity after a 12-month strength training intervention. Together, it seems that different types of exercise training are able to induce cognitive improvements that are mediated by brain functional and structural adaptations in elderly persons. Nonetheless, previous studies focused on brain functional adaptations during the performance of cognitive tasks that can be employed with fMRI measurements, whereas our study provides first insight to exercise training induced brain functional adaptations during a challenging treadmill walking task. These adaptations were correlated with cognitive improvements in the elderly participants.

In the present study, improved cognitive performance was mainly (in eight of nine instances) related to a *reduction* in PFC activity during walking. This finding is consistent with Erickson et al. (2007a) who performed a cognitive training intervention including five 1-h sessions. They reported a training induced reduction of brain activity in most regions that displayed activity during cognitive dual-tasking. One exemption was a training-related *increase* in activity of the bilateral dorsolateral PFC that was correlated with better performance. The authors explained this phenomenon with a switched strategy to perform dual-task-related activities resulting in the recruitment of other brain areas. A similar mechanism could have become evident in our own study where only one task of executive function (the Stroop Word-Color Interference task) was associated with a training-induced increase in (right PFC) activity. Similarly, an fNIRS study with older adults by Hyodo et al. (2012) demonstrated enhanced right frontopolar activity during the Stroop task when performed after an acute bout of 10 min ergometer cycling at moderate intensity. Enhanced right frontopolar brain activity was also associated with improved Stroop Interference results. Furthermore, another recent fNIRS study by Dupuy et al. (2015) found that young and old women with higher aerobic fitness showed increased brain activity in the right inferior frontal gyrus during the Stroop task.

Interestingly, in the three tests assessing different components of executive function (TMT-B, Stroop Word-Color, and Executive Control tasks) correlation was evident exclusively with either left or right hemispheric PFC activity changes, whereas processing speed (TMT-A) was correlated with bilateral PFC activity reductions. Measures of processing speed were shown to be associated with age-related global white matter deterioration (fractional anisotropy), but not to specific brain regions (Kuznetsova et al., 2015). Moreover, processing speed was associated with myelin integrity, particularly in the prefrontal lobe and the genu of corpus callosum which represent late-myelinating regions in brain development and are, therefore, highly vulnerable to breakdown in normal aging (Lu et al., 2011). These studies may explain why in the present study improvements in TMT-A were not specifically correlated with either left or right hemispheric PFC activity reductions, but rather to bilateral adaptations.

### Strengths and limitations

Strengths of the present study were the experimental design of the fNIRS treadmill protocol where we included and averaged several repetitions of the two walking conditions in order to minimize bias from potential learning or accommodation effects. Additionally, we used a multi distance fNIRS instrument, which eliminates measurement bias from skin blood flow changes (Jung et al., 2015). Likewise, some researchers questioned the outcome of previous studies that applied other fNIRS methods and concluded that in fact not PFC oxygenation was measured but rather superficial blood flow in skin and muscle caused by cardiovascular activity during exercise (Miyazawa et al., 2013; Jung et al., 2015). The following limitations have to be acknowledged. We focused fNIRS assessments on left and right PFC activity during walking due to its association with cognitive executive functions. However, for future investigations it would be interesting to obtain data from other brain areas related to walking and their functional adaptations to exercise training. Furthermore, our correlation analyses of changes in brain function during walking and cognitive behavioral outcomes do not necessarily imply causality. Nonetheless, the outcome of this correlation still provides valuable information since the changes in the variables were observed between two distinct time points before and after a defined exercise training program, whereas in cross-sectional studies it is unknown at what time in the past the changes occurred (Salthouse, 2011). What can be seen as another possible limitation of our study is the difference in age between the two intervention groups. However, it is well-known that cognitive decline cannot be interpreted based on chronological aging *per se* but is rather affected by several biological and physical health domains as well as environmental and genetic factors (MacDonald et al., 2011; DeCarlo et al., 2014; Walhovd et al., 2014). Therefore, and considering that the physical and cognitive baseline values were not different, with the exception of one cognitive task, we argue that the age difference between the two intervention groups did not have an impact on our results. Finally, a passive control group might have helped to control bias from repeated testing. However, due to the aforementioned fNIRS test procedure that included many averaged repetitions of the same test condition at pre- and post-test assessment, we could reduce this sort of bias to a minimum.

## Conclusions

The present study demonstrated three mechanisms of exercise training induced functional brain plasticity during treadmill walking in elderly participants who underwent 8 weeks of interactive cognitive-motor video game dancing or conventional balance training. These mechanisms comprise (1) a bilateral reduction in prefrontal brain activity at preferred and fast locomotion speed (with larger effects in the video game dance group), (2) an increase in hemispheric PFC activity asymmetry, and (3) an increased differentiation in PFC activity related to walking speed. The adaptations resemble more trained or young adult-like brain functions as observed in previous cognitive training interventions and cross-sectional fMRI and fNIRS studies on brain activity in cognitive and walking tasks, respectively. The prefrontal adaptations were correlated with improved performance in executive functions and processing speed. These novel findings imply that exercise training is able to reduce the need of prefrontal resources of executive function and attention involved in challenging treadmill walking. We speculate that the elderly might benefit from these additional cognitive resources to focus their attention on other processes while walking. This would be of practical importance in attention demanding real-life situations such as crossing streets or walking while talking and could potentially reduce the risk of falling. Future investigations are warranted that should focus on additional brain areas involved in locomotion and that should include other types of exercise training and challenging walking conditions in order to substantiate or refute the presented findings.

## Author contributions

PE: study preparation and conception, participants' recruitment, data acquisition, statistical analysis, data interpretation, drafting manuscript. MW: study conception, technical advice on fNIRS, data interpretation, critically revising manuscript. MS: study preparation and conception, participants' recruitment, training instruction, data acquisition, critically revising manuscript. Ed: study conception, data interpretation, critically revising manuscript. All authors read and approved the final manuscript.

## Funding

This work was supported by the Zürcher Kantonalbank within the framework of sponsoring of movement sciences, sports and nutritional sciences at ETH Zurich. Zürcher Kantonalbank had no influence on the study design and the analyses presented in this paper, had no access to the data, and did not contribute to this manuscript in any way.

### Conflict of interest statement

The authors declare that the research was conducted in the absence of any commercial or financial relationships that could be construed as a potential conflict of interest.
